# A novel trait to reduce the mechanical damage of peach fruits at harvest: The first genetic dissection study for peduncle length

**DOI:** 10.1007/s11032-025-01547-3

**Published:** 2025-02-24

**Authors:** Cassia da Silva Linge, Angelo Ciacciulli, Irina Baccichet, Remo Chiozzotto, Elisa Calastri, Alessandro Giulio Tagliabue, Laura Rossini, Daniele Bassi, Marco Cirilli

**Affiliations:** 1 Department of Agricultural and Environmental Sciences - Production, Landscape, Agroenergy, University of Milan, Milan, Italy; 2 Council for Agricultural Research and Economics (CREA), Research Centre for Olive, Fruit and Citrus Crops, Acireale, Italy

**Keywords:** 9 K SNP array, *Prunus persica* L. Batsch, Bi-parental Mapping, Quantitative trait loci, Fruit physical injuries, Breeding

## Abstract

In peach, a long peduncle can help minimize mechanical damages/physical injuries in the fruit at harvest and can also be useful in postharvest handling and transportation. In view of genetically dissecting the peduncle length (PL) in peach, we have performed a Quantitative Trait Locus (QTL) mapping study for PL using a F_2_ progeny of 117 individuals from the cross ‘PI 91459 [NJ Weeping]’ x ‘Bounty’ (WxBy). The progeny was phenotyped for three years (2011, 2012 and 2014) and the QTL mapping analysis was performed using four methods: Kruskall-Wallis, Interval Mapping, Multiple QTL Mapping and Genome-Wide Composite Interval Mapping. QTL analysis led to the identification of 9 QTLs distributed on linkage groups (LG) 1, 2, 4, 5, 6 and 7. A stable QTL was identified on LG6 (22,978,897 to 24,666,094 bp) and explained up to 63% of the phenotypic variance. Within the genetic interval of the stable QTL on LG6 potential candidate genes with functional annotation encompassing cellular expansion, hormone regulation, transcriptional regulation, developmental processes such as meristem development, and responses to environmental cues were found. The results reported in this study represent the first insight into the genetic basis of PL and a step forward towards the introduction of novel traits in peach commercial breeding in order to minimize the problems related to mechanical damage/injuries to peach fruits that commonly might occur during at harvest and post-harvest processes.

**Supplementary Information:**

The online version contains supplementary material available at 10.1007/s11032-025-01547-3.

## Introduction

**Fig. 1:**
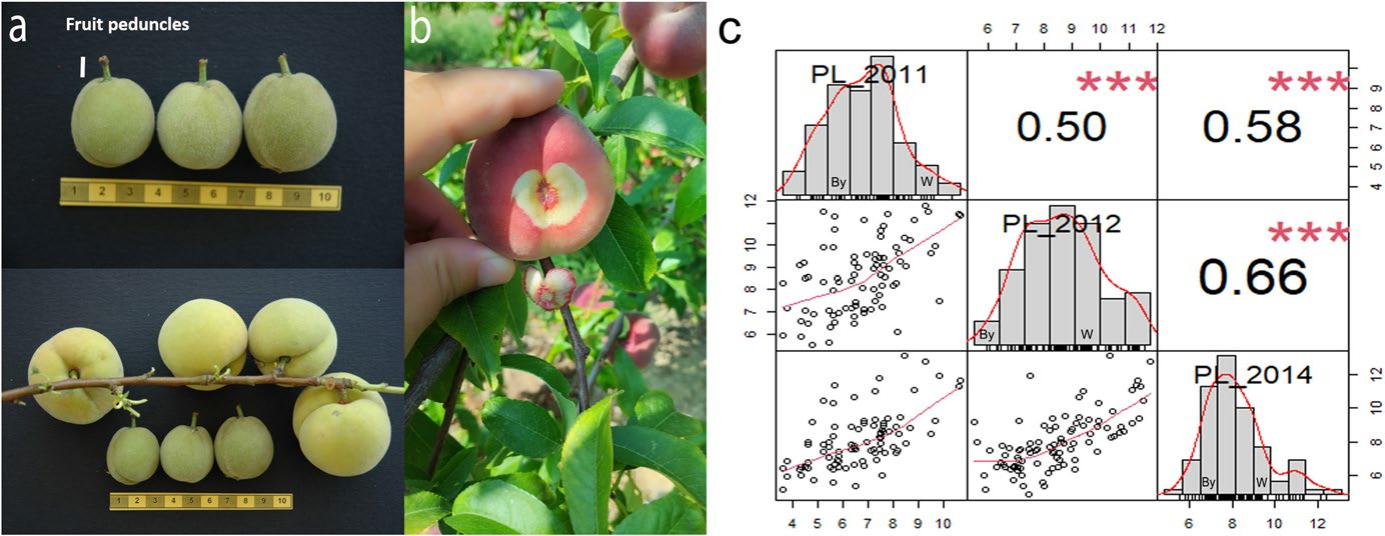
(**a**) Illustration of fruit peduncles; (**b**) Illustration of physical injuries in peach fruit during the harvest; (**c**) Distribution of PL in the 117 WxBy seedlings and the grandparents NJ Weeping (W) and Bounty (By) (on the diagonal); bivariate scatter plots with a fitted line (on the bottom of the diagonal); correlations plus the significance level (*p* < 0.001) of PL and years in the WxBy seedlings in 2011, 2012 and 2014 (on the top of the diagonal)

Peach is one of the most important temperate fruit crops, with a global production reaching approximately 26.3 million tons (FAOSTAT 2020). According to FAOSTAT, the largest worldwide producers were China, Spain, Italy, Turkey and Greece. The low sensory quality of the fruits often significantly impacts consumer acceptance, which results in a static or decreasing peach consumption in Europe and the United States (Iglesias and Echeverría 2009; Minas et al. 2023). The main attributes driving consumer preferences are visual appearance and aroma (Olmstead et al. 2015). Fruit visual appearances involve traits such as size, shape, color and mechanical damages/physical injuries (Li et al. 2016; Masuda et al. 2023). The physical injuries in peach fruits primarily stem from improper physical handling at harvest, due to short peduncle length causing the fruit being pressed to the branch (Fig. 1). Fruits are highly susceptible to mechanical damage during this process, leading to structural, tissue, and cell damage caused by impact, compression, abrasion, puncturing, testing, or a combination of these actions. These damages may also increase susceptibility to decay (Li and Thomas 2014). The presence of a longer fruit peduncle reduces the likelihood of contact between the branch and the fruit during the final swell, facilitates easier fruit separation from the plants, preventing potential skin damage at harvest and can also be beneficial in postharvest handling and transportation. Despite the importance of this trait, to our knowledge, only one study related to the peduncle has been previously reported in peach (Bassi and Rizzo 2002). In that study, the authors concluded that a long fruit peduncle might allow the fruit to hang without touching the branch. Thus, peduncle length (PL) could be an interesting novel trait to be introduced in peach breeding programs.

The study of fruit PL has been performed in several species with different goals. In apples, the presence of a peduncle represents a quality indicator, and its absence can be considered a criterion for discarding fruits before storage, according to a good harvesting and postharvest practices study. (Abi Tarabay et al. 2018). In sweet cherry, green peduncles speak for fruit freshness and are crucial parameters for consumers guiding their purchasing decisions (Linke et al. 2010). The PL trait was used for differentiation of *Plinia cauliflora*, *P. trunciflora*, and *P. jaboticaba* species (Danner et al. 2011). In myrtle, PL over 2 cm was linked to easy fruit detachment by both hand and machine harvest (Medda and Mulas 2021). Similar results were observed in cucumber, where a successful harvesting process depends on a long PL (Song et al. 2016). The absence of peduncles was associated with fruits more wilted in a postharvest quality study of Jaboticaba (Nascimento et al. 2013).

The Quantitative Trait Locus (QTL) mapping approach is widely used for genetically dissect complex traits. This approach involves the use of bi-parental populations where the parents should exhibit contrasting characteristics for the target trait. (Taliei et al. 2024). Several methodologies for QTL mapping are available. The interval mapping (IM) is based on the segregation information of pairs of adjacent markers using the maximum likelihood method to estimate the frequency of recombinants and the magnitude of the effect of the QTL in the interval between two linked markers (Lander and Botstein 1989). However, the drawback of this method is that other QTLs outside of the interval are not considered. To deal with this issue, composite interval mapping which uses a multiple regression model has been proposed (Zeng 1993). Subsequently, based on CIM, a series of multi-QTL mapping models has been released. The multiple-interval mapping (MQM) incorporates epistasis into the model, considers multiple intervals simultaneously and uses markers as cofactors in an approximate multi-QTL model with all loci acting additively to each other (Jansen 1993, 1994; Jansen and Stam 1994). Although, these methods significantly improved the studies involving QTL mapping approaches, the detection of small effects QTLs and closely linked QTLs remains challenging. Thus, a genome-wide composite interval mapping (GCIM) has been recently proposed (Wang et al. 2016; Wen et al. 2019; Zhang et al. 2020). Firstly, GCIM uses a single-locus random-SNP-effect mixed linear model in genome-wide association studies (GWAS) to identify putative QTL on the genome. Secondly, polygenic variance in GWAS is estimated. Afterwards, all the peaks in the negative logarithm P-value curve against genome position for each QTL effect are viewed as potential QTLs and these potential QTLs are placed into a multi-locus genetic model for true QTL identification (Zhang et al. 2020).

Several studies using QTL mapping approach for genetically dissecting complex traits have been performed in peach (Pirona et al. 2013; da Silva Linge et al. 2015; Abdelghafar et al. 2020; da Silva Linge et al. 2024; Eduardo et al. 2011; Pacheco et al. 2014; Nuñez-Lillo et al. 2019; Rawandoozi et al. 2021; Hernández Mora et al. 2017; Romeu et al. 2014). However, there are no studies focusing on understanding the genetic basis of PL. Thus, the objectives of this study were to: emphasize the importance of PL as a novel trait to be considered in peach breeding programs; perform the first QTL mapping study for PL using an F_2_ progeny from a cross between an ornamental peach with long peduncles and a commercial cultivar and carry out a candidate gene analysis based on the annotation of the peach genome v2.0 within the genetic interval of the potential stable QTLs detected.

## Material and methods

### Plant material

An F_2_ progeny of 117 individuals (WxBy) was obtained from self-pollination of an F_1_ seedling from the cross ‘PI91459’ (NJ Weeping) x ‘Bounty’. ‘NJ Weeping’ is a late blooming and ripening ornamental peach with a long fruit peduncle. ‘Bounty’ is a medium season peach with a standard (short) fruit peduncle.

Individuals of the F_2_ progeny were located in in Faenza (Emilia Romagna region, lower Po valley, northern Italy). The trees were planted with a spacing of 1 m within and 4 m between rows. The WxBy progeny segregates for maturity date (Pirona et al. 2013), fruit weight and size (da Silva Linge et al. 2015) and fruit peduncle length.

### Phenotypic data

We collected 10 fruits per seedling at commercial ripening across three years (2011, 2012 and 2014). In view of collecting fruits in similar commercial ripening stage, an Index of Absorbance Difference (I_AD_) of 0.2–0.5, were adopted. IAD values were estimated as the average value read for each fruit cheeks by a DA-meter portable spectrometer (Sinteleia S.r.l., Bologna, Italy). The peduncles were removed from each fruit, and their PL was measured by a digital caliper. A Welch’s t-test was employed to verify the significant differences between PL means of ‘NJ Weeping’ and ‘Bounty’. Additionally, we analyzed the Spearman correlation between the years using the Performance Analytics R package (Peterson et al. 2014). The broad and narrow sense heritability was estimated considering the three years (2011, 2012 and 2014) using the R package Sommer (Covarrubias-Pazaran 2016) and the *vpredict* function:

$$vpredict (object, transform)$$

where: object represents a model fitted with the mmer function and transform is the formula to calculate the function. For broad sense heritability, we used the following model:

$$model<-mmer(Trait\sim Year,$$

$$random=\sim vsr(Seedlings),$$

$$rcov=\sim vsr\left(dsr\left(Year\right),units\right)$$

with:

$${h}^{2}=\frac{VG}{VP}$$

where “VG” is the total genetic variance and “VP” is the phenotypic variance.

For narrow sense heritability, we used:

$$mix<-mmer(Trait\sim Year,$$

$$random=\sim vsr(Seedlings,Gu=K),$$

$$rcov=\sim vsr\left(dsr\left(Year\right),units\right)$$

where “K” refers to the additive relationship matrix. The formula included in the function was:

$${h}^{2}=\frac{VA}{VP}$$

where “VA” is the additive genetic variance and “VP” is the phenotypic variance.

### QTL mapping

For the QTL mapping analysis, we used the previously published WxBy linkage map (Supplementary Table 1) comprising 877 SNPs, with an average marker distance of 0.52 cM and covering 93.6% of the physical length of the peach genome (da Silva Linge et al. 2015). Initially, the analysis was carried out with the MapQTL software (Van Ooijen 2009) using three methods. The non-parametric Kruskal–Wallis (Khan et al.^2013^) rank sum test was used to search phenotype–marker associations without assuming a normal distribution of phenotypic data. In order to obtain an overall significance level of about p = 0.05, a stringent significance level of p = 0.001 was adopted as threshold for the detection of a QTL for the individual test. The Interval Mapping method (IM) and Multiple QTL Mapping (MQM) were also employed. For genome wide analysis, the significant LOD thresholds were calculated by random permutation test (with 10,000 replicates) at the α = 0.05 level. The detection of QTLs was performed via IM with 95% significance (p > 0.05) to identify QTLs with significant main effects. The MQM method was used to detect possible QTLs masked by QTLs identified by IM. Applying the option "automatic cofactor selection" in the MQM strategy, different cofactor markers were tested around the putative QTL positions selecting as final cofactors the closest marker to each QTL. The results referent of the percentage of variation explained by each QTL (R^2^) as well as the total variance explained by all the QTLs affecting a trait were obtained in the final MQM model.

The R^2^ was calculated as:

$$100\times \frac{(H0\_var-var)}{\text{Phenotypic variance of the population}}$$

where H0_var is the residual variance under the current null hypothesis considering all the QTLs and cofactors mapped for the trait.

Subsequently, the genome-wide composite interval mapping (GCIM) method was applied using the QTL.gCIMapping R package (Zhang et al. 2020). The default settings were used with a minimum LOD threshold score of 3.0 and significance level, (*P* ≤ 0.05) was used to identify significant QTLs. In the GCIM method, the R^2^ was calculated as:

$$\frac{\text{Genetic variance explained by the QTL} }{\text{Total phenotypic variance}}\times 100$$

In this work, we considered a QTL as “stable” when QTLs exhibited a significant effect and were located in overlapping positions across at least two years using the same or different method. The QTLs were named as “q” + trait name abbreviation + abbreviation of the population + scaffold + number of the chronological QTL for this trait reported on this chromosome (e.g. *qPL_WBy_4.1*). Graphical representation of QTLs on the WxBy linkage map were generated with MapChart 2.3 software (Voorrips 2002).

### Haplotype analysis

Haplotype analysis was carried out within the genetic interval of the stable QTL identified on LG6. The comparison among phenotypic values of each segregating class was estimated by Welch ANOVA and Games Howell post-hoc tests. Statistical analyses were performed using the ggstatsplot R package (Patil 2021).

### Candidate genes

Candidate gene searches were performed based on the annotation of peach genome v2.0 for the genetic interval of the stable QTL located on LG6. Complete coding sequences were obtained from peach genome v2.0 assembly (Verde et al. 2017) on Genome database for Rosaceae-GDR (Jung et al. 2018). Similarity searches for the coding sequences were conducted using blastn against NCBI.

## Results

### Phenotypic data

The Grandparents ‘NJ Weeping’ and ‘Bounty’ exhibited, according to Welch’s t-test (*p* < 0.0001), significantly different values for PL in the three years evaluated (Supplementary Table 2). ‘Bounty’ is characterized by a standard (short) fruit peduncle, while ‘NJ Weeping’ revealed peduncle length approximately 1.7 × larger in 2011 and 2012 and 1.4 × in 2014 (Supplementary Table 2) than fruits of Bounty. Regarding the WxBy F_2_ progeny, the PL ranged from 3.6 mm to 10.6 mm with an average of 6.6 mm in 2011. In 2012, the PL values varied from 5.5 mm to 11.8 mm with an average of 8.5 mm. In 2014, the values ranged from 4.9 to 13.1 with an average of 8.0 mm (Supplementary Table 2 e 3). The estimated broad sense heritability for PL considering the three years was 0.59, while the narrow sense heritability was 0.52 (Supplementary Table 2). Comparisons of PL between years resulted in significant correlations, with the highest coefficient (0.66) observed in 2012 and 2014 (Fig. 1c).

### QTL mapping

The non-parametric Kruskal–Wallis test revealed SNPs correlated with PL on LGs 3, 4, 5 6 and 7 across the three years evaluated (Supplementary Table 4). In 2011, the SNPs correlated with PL were mainly located on LG4 (115) with the highest K value (31.4), observed in SNP_IGA_413115 (LG4: 11,593,768 bp). In addition, 64 significantly correlated SNPs with PL were also detected on LG6 (K values ranging from 14.1 to 22.9) and three SNPs on LG7 (K varying from 14.0 to 14.1). Regarding 2012, a total of 106 correlated SNPs with PL were associated with PL on LG6. The highest K value (49.8) was detected in SNP_IGA_680857 (LG6: 24,155,317 bp). Moreover, SNP_IGA_597728 (LG5: 13,862,304 bp), SNP_IGA_754512 (LG7: 9,446,098 bp) and SNP_IGA_754563 (LG7: 9,451,629 bp) were significantly correlated with PL demonstrating K values of 14.5 and 14.6, respectively. In 2014, the number of correlated SNPs with PL were 115. Among them, 109 were located on LG6 with the highest K value (70.8) in SNP_IGA_680615 (LG6: 24,117,844 bp). Additional six SNPs on LG3 were also identified with K values ranging from 14.9 to 18.0 (Supplementary Table 4).

**Fig. 2:**
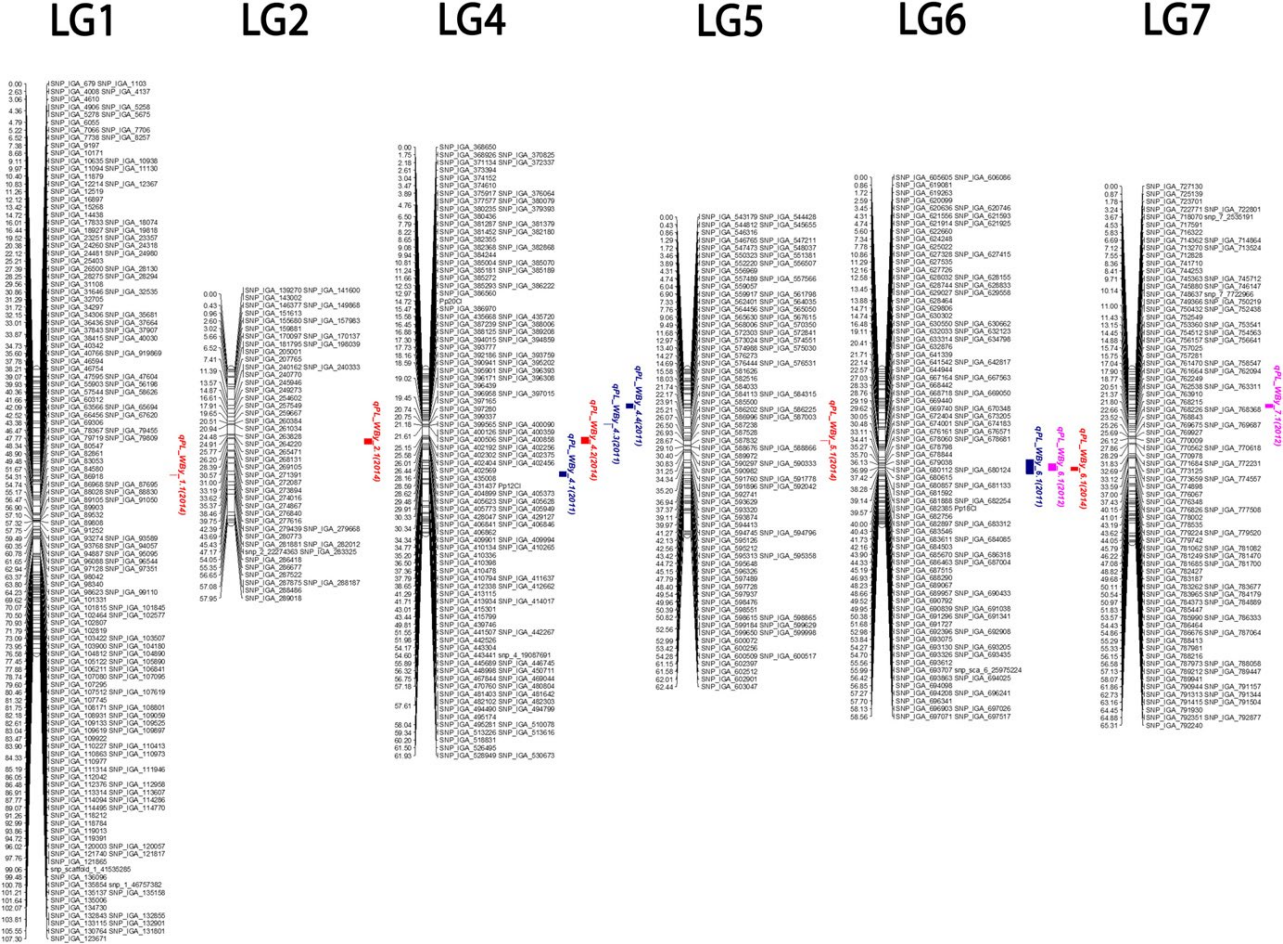
Mapped QTLs controlling PL by MQM and GCIM. Marker names are listed at the right side of each LG, and the genetic distances (in centimorgans) are listed at the left of each LG. QTLs are drawn at the right of each LG and are represented as central bar. QTLs for PL in different years are represented with different bar fills: blue for 2011, pink for 2012 and red for 2014

In the QTL analysis using IM and MQM methods, permutation tests revealed LOD thresholds for detecting potential QTLs at the genome-wide level of 3.5 in 2011 and 3.6 in 2012 and 2014 (Table 1). Consequently, we considered a QTL significant when it showed LOD values greater than 3.6. Following this criterion, we mapped two QTLs associated to PL in 2011 and 2012 and four in 2014 (Table 1; Fig. 2). QTLs mapped for PL accounted for approximately 41, 54 and 77% of the phenotypic variation (PV) observed in 2011, 2012 and 2014, respectively. The *qPL_WBy_6.1*, was stable across the three years and explained up to 63% of PV. In 2011, the *qPL_WBy_4.1* revealed the highest LOD score (9.82) and explained 23.3% of PV. The *qPL_WBy_7.1* located at LG7 was mapped only in 2012, explained approximately 10% of the PV and the LOD peak was detected near the marker SNP_IGA_754563 (9,451,629 bp). Three QTLs (*qPL_WBy_4.2*, *qPL_WBy_1.1*, *qPL_WBy_2.1*) were mapped only in 2014. The *qPL_WBy_4.2*, explained approximately 6% of PV and peaked at SNP_IGA_402404 (8,547,997 bp), while *qPL_WBy_1.1* and *qPL_WBy_2.1* explained approximately 4% of PV each and peaked at SNP_IGA_55903 (16,864,858 bp) and SNP_IGA_269105 (20,695,265 bp), respectively.

**Table 1:** Mapped QTLs in WxBy F_2_ progeny by Interval mapping (IM) and Multiple QTL mapping (MQM): trait, permutation test, QTL name, year mapped, linkage group (LG), start and final position of the QTL in WxBy map, cofactor marker, genomic position of cofactor marker, LOD and variations explained (R^2^); stable QTLs are in bold

Trait	Permutation test	Multiple QTL mapping								
	Genome-wide threshold *p* < 0.05	QTL^a^	Year	LG	Start^b^	Final^b^	Cofactor marker	Genomic position^c^	LOD	R^2^
Peduncle Length (PL)	2011 = 3.5	*qPL_WBy_4.1*	2011	4	38.65	40.65	SNP_IGA_412338	11,208,347	9.82	23.3
		***qPL_WBy_6.1***	**2011**	**6**	**34.10**	**39.57**	**SNP_IGA_682756**	**24,666,094**	**7.93**	**18.0**
	2012 = 3.6	***qPL_WBy_6.1***	**2012**	**6**	**37.42**	**38.28**	**SNP_IGA_681592**	**24,295,392**	**15.3**	**43.5**
		*qPL_WBy_7.1*	2012	7	13.15	14.45	SNP_IGA_754563	9,451,629	4.84	10.5
	2014 = 3.6	***qPL_WBy_6.1***	**2014**	**6**	**36.99**	**37.42**	**SNP_IGA_680615**	**24,117,844**	**32.14**	**63.8**
		*qPL_WBy_4.2*	2014	4	25.58	26.01	SNP_IGA_402404	8,547,997	5.41	5.7
		*qPL_WBy_1.1*	2014	1	39.93	39.93	SNP_IGA_55903	16,864,858	4.05	4.1
		*qPL_WBy_2.1*	2014	2	26.20	28.39	SNP_IGA_269105	20,695,265	3.67	3.7

^a^QTLs were named as “q” + trait name abbreviation + abbreviation of the population + scaffold + number of the chronological QTL for this trait reported on this chromosome

^b^Genomic interval of the QTL in cM

^c^Genomic position of cofactor marker in bp (based on the peach genome v2.0)

In order to detect potential QTLs with small effects and those closely linked, we also carried out QTL analysis using the Genome-wide Composite Interval Mapping (GCIM) method. The QTLs mapped using the GCIM method is shown in Table 2. The method GCIM enabled the identification of two additional QTLs (*qPL_WBy_4.3* and *qPL_WBy_4.4)* on LG4 in 2011 and one small effect QTL (*qPL_WBy_5.1*) on LG5 in 2014 (Table 2; Fig. 2). QTLs previously mapped using IM and MQM (*qPL_WBy_1.1, qPL_WBy_4.1, qPL_WBy_4.2 and qPL_WBy_6.1*) were also identified using the GCIM method. However, slightly different LOD scores and R^2^ were observed (Table 1 and 2).

**Table 2:** Mapped QTLs in WxBy F_2_ progeny by genome-wide composite interval mapping (GCIM): Trait, QTL name, year mapped, linkage group (LG), start and final position of the QTL in WxBy map, left and right markers, genomic interval, LOD and variations explained (R^2^); previously mapped QTLs using IM and MQM are in bold

Trait	Genome-wide composite interval mapping									
	QTL^a^	Year	LG	Start^b^	Final^b^	Left marker	Right marker	Genomic interval^c^	LOD	R^2^
Peduncle Length (PL)	***qPL_WBy_6.1***	**2011**	**6**	**34.41**	**34.41**	**SNP_IGA_678060**	**SNP_IGA_678060**	**22,978,897**	**7.54**	**12.54**
	*qPL_WBy_4.3*	2011	4	20.74	20.74	SNP_IGA_397280	SNP_IGA_397280	6,556,594	4.22	5.44
	***qPL_WBy_4.1***	**2011**	**4**	**38.65**	**41.30**	**SNP_IGA_412338**	**SNP_IGA_413115**	**11,208,347—11,593,768**	**4.40**	**4.30**
	*qPL_WBy_4.4*	2011	4	12.97	14.72	SNP_IGA_386560	Pp20Cl	4,197,626—5,483,781	3.03	2.10
	***qPL_WBy_6.1***	**2012**	**6**	**35.70**	**36.13**	**SNP_IGA_678844**	**SNP_IGA_679038**	**23,384,329—23,447,467**	**15.09**	**32.71**
	***qPL_WBy_6.1***	**2014**	**6**	**37.42**	**38.28**	**SNP_IGA_680615**	**SNP_IGA_680857**	**24,117,844—24,155,317**	**33.11**	**62.34**
	***qPL_WBy_4.2***	**2014**	**4**	**26.44**	**28.16**	**SNP_IGA_402569**	**SNP_IGA_435008**	**8,581,271—10,635,613**	**3.10**	**1.92**
	***qPL_WBy_1.1***	**2014**	**1**	**39.93**	**39.93**	**SNP_IGA_55903**	**SNP_IGA_55903**	**16,864,858**	**3.56**	**1.61**
	*qPL_WBy_5.1*	2014	5	26.93	26.93	SNP_IGA_587528	SNP_IGA_587528	9,908,867	3.28	0.89

^a^QTLs were named as “q” + trait name abbreviation + abbreviation of the population + scaffold + number of the chronological QTL for this trait reported on this chromosome

^b^Genomic interval of the QTL in cM

^c^Genomic interval of the QTL in bp (based on the peach genome v2.0)

**Table 3:** Predicted candidate genes for Peduncle Length (PL) in the genetic interval of the stable QTL on LG6, *qPL_WBy_6.1*, (22,978,897 to 24,666,094 bp)

Predicted Genes on GDR	NCBI predicted genes	Source	Functional Annotation	Identity (E-value)
*Prupe.6G226100*	LOC18773652	*Prunus persica*	Indole-3-acetic acid-amido synthetase Gretchen Hagen 3 (GH3)	100% (0.0)
*Prupe.6G231500*	LOC18773759	*Prunus persica*	Ethylene-responsive transcription factor (ERF)	100% (0.0)
*Prupe.6G233100*	LOC18774963	*Prunus persica*	UDP-glycosyltransferase (UDG)	100% (0.0)
*Prupe.6G234000*	LOC18774805	*Prunus persica*	Pentatricopeptide repeat-containing protein (PPR)	100% (0.0)
*Prupe.6G238200*	LOC18774511	*Prunus persica*	WD repeat-containing protein (WDR)	100% (0.0)
*Prupe.6G239900*	LOC18772400	*Prunus persica*	Pentatricopeptide repeat-containing protein (PPR)	100% (0.0)
*Prupe.6G241900*	LOC18772943	*Prunus persica*	LRR receptor-like serine/threonine-protein kinase (LRR-RLK)	100% (0.0)
*Prupe.6G242400*	LOC18773658	*Prunus persica*	AP2-like ethylene-responsive transcription factor TOE3	100% (0.0)
*Prupe.6G244300*	LOC18772322	*Prunus persica*	WRKY transcription factor (WRKY)	99.7% (0.0)

### Haplotype analysis

The SNP haplotype analysis performed within the genetic interval of the stable QTL identified on LG6 (*qPL_WBy_6.1;* 22,978,897 to 24,666,094 bp), revealed four common haplotypes among 103 out of 117 individuals from WxBy progeny (Supplementary Table 5). WxBy segregated 1:2:1 (chi-square = 0.35) for the H1, H2H3, and H4 haplotypes, respectively. The comparison among phenotypic values of each haplotype revealed significant differences with the haplotype H1 being associated with a higher values of PL, while the H4 with the lowest PL values (Supplementary Fig. 1). Individuals carrying the H1 haplotype had average PL values of 7.60 mm, 10.05 mm, and 10.19 mm in 2011, 2012, and 2014, respectively. In contrast, individuals with the H4 haplotype exhibited average PL values of 5.30 mm, 7.12 mm, and 6.38 mm across the same years. Individuals with H2H3 haplotype revealed significantly highest values of PL in comparison to H4 (averages of 6.62, 8.43 and 7.82, respectively).

### Candidate genes

Considering the stability across three years and QTL detection methods, we decided to further focus on the genomic region underlying the QTL on LG6 to search for putative candidate genes. In total, 954 genes were annotated within the confidence interval of stable QTL on LG6 (*qPL_WBy_6.1*; 22,978,897 to 24,666,094 bp), based on peach genome annotation v2.0 (Supplementary Table 6). Among them, nine annotated genes (*Prupe.6G226100*, *Prupe.6G231500, Prupe.6G233100*, *Prupe.6G234000*, *Prupe.6G238200, Prupe.6G239900*, *Prupe.6G241900, Prupe.6G242400* and *Prupe.6G244300)* encoded the following proteins with potential roles in peduncle development: indole-3-acetic acid-amido synthetase Gretchen Hagen 3 (GH3), ethylene-responsive transcription factor (ERF), UDP-glycosyltransferase (UGT), pentatricopeptide repeat-containing protein (PPR), WD repeat-containing protein (WDR), LRR receptor-like serine/threonine-protein kinase (LRR-RLK), AP2-like ethylene-responsive transcription factor TOE3 and WRKY transcription factor (WRKY TFs) (Table 3). Based on literature searches, these genes could be involved in developmental processes, stress responses, hormone regulation, gene expression regulation, and cellular signaling, all of which directly or indirectly affect fruit peduncle length/elongation.

## Discussion

The introduction of novel traits is crucial for achieving the main objective of breeding programs, considering that only a few traits are typically taken into account by breeders when planning crossings (Castle et al. 2006). Fruit peduncles length (PL) could be an interesting trait for breeders to be introduced into commercially cultivars due to its importance in reducing the mechanical damage to peach fruits during harvest. To gain deeper understanding of the genetic mechanisms underlying PL, we phenotyped an F_2_ progeny (WxBy) over three years and observed transgressive segregation (Supplementary Table 2). The broad sense heritability (0.59) was rather high and consistent with the unique reported study for PL in peach, where the value was 0.56 (Bassi and Rizzo 2002). Significant correlations between years (up to 66%) suggested a consistent pattern or trend of PL over time, indicating a degree of stability in how this trait behaves across different years (Fig. 1c). In addition, the first QTL mapping study for PL was successfully performed.

Concerning the QTL mapping methods applied, although the KW method did not precisely pinpoint the location of the QTLs, especially in 2012 and 2014, where we observed the associated markers to PL spread across the entire LG6 (Supplementary Table 4), the highest K values still corresponded to the SNPs identified as cofactor by the MQM method in 2012 and 2014, respectively. MQM and GCIM methods successfully mapped the QTLs, precisely determined their location and the intervals on the LGs, and provided the information about the phenotypic variance explained by each QTL (Table 1 and 2). The CGIM method enabled the identification of stable QTLs and also detected the small-effect QTLs on LG4 and 5 (*qPL_WBy_4.3, qPL_WBy_4.4* and *qPL_WBy_5.1*) that could not be mapped using the IM and MQM methods (Table 2). Based on the findings of this study, we recommend using GCIM due its ability to identify both larger and small-effect QTLs. Regarding the stable and large-effect QTL on LG6 (*qPL_WBy_6.1*), the detectability of the QTL was consistent across the three methods used (IM, MQM, and GCIM), indicating that these QTLs are robustly detectable regardless of the method employed.

Further investigations within the genetic interval of the stable QTL *qPL_WBy_6.1* (22,978,897 to 24,666,094 bp on peach genome v2.0), enabled the identification of SNP haplotype (H1) associated with higher values of PL (inherited from Weeping). In addition, the candidate genes analysis revealed genes with functional annotation related to cellular expansion, hormone regulation, transcriptional regulation, developmental processes such as meristem development, and responses to environmental cues (Table 3). GH3 family genes encode enzymes that play a central role in the auxin signaling transduction pathway. These enzymes conjugate auxin to amino acids regulating its activity and homeostasis. GH3 genes have been previously associated with PL in cucumber and cowpea (Song et al. 2016; Lo 2019). In turn, ERF family genes play an important role in several developmental processes and stress tolerance (Zhuang et al. 2021). Previous studies have shown that ERF function affects PL in rice (Zhang et al. 2022). In litchi (*Litchi chinensis*), reduced ERF expression decreased the fruit abscission while concurrently enhancing pedicel/peduncle growth (Yi et al. 2021). UDP-glycosyltransferases and glycosyltransferases are involved in plant growth, development, hormone regulation, and cell wall biosynthesis (Li et al. 2001; Hou et al. 2004; Farrokhi et al. 2006). These enzymes could indirectly influence peduncle elongation through their roles in cell wall biosynthesis and hormone regulation. Glycosyltransferases have been previously involved with peduncle elongation in rice (Kandpal et al. 2020). Genes encoding PPR proteins were also identified within the QTL interval. PPR proteins constitute one of the largest and most complex families in plants, playing crucial roles in the post-transcriptional regulation of organelle gene expression. In rice, a mutant named *sped1-D*, characterized by shortened pedicels and/or secondary branches, was previously identified. The *sped1-D* gene responsible for this phenotype was cloned and found to encode a PPR protein involved in the gibberellin signaling pathway (Jiang et al. 2014). This finding suggests that PPR proteins may be involved in the regulation of pedicel and peduncle elongation, potentially through their influence on mitochondrial and chloroplast function, which in turn affects cellular energy production and hormone regulation essential for plant growth. A gene functionally annotated as WD repeat-containing protein influenced the petiole length and diameter in cucumber (Li et al. 2024). Additionally, we identified Leucine-rich repeat receptor-like serine/threonine-protein kinase (LRR-RLK) genes within the target interval. LRR-RLK are the largest group of receptor-like kinases in plants and play important roles in development and stress responses (Liu et al. 2017). In tomato, the LRR-RLKs, particularly ERECTA, are crucial in regulating stem elongation, as evidenced by their influence on short internodes and pedicels (Zhao et al. 2023). WRKY TFs are critical for numerous processes of plant growth and development, from stem elongation, embryogenesis, and trichome development to senescence (Li et al. 2021). Thus, WRKY TFs might modulate the expression of genes involved in cell elongation, division and hormonal pathways, which are fundamental processes contributing to peduncle elongation. In *Rosa hybrida*, WRKY TFs exhibit significant upregulation in response to peduncle necking (Lear et al. 2022). Interestingly, *Prupe.6G242400* is also located within the genetic interval of the stable QTL for PL on LG6. Previous studies have identified a dominant floral mutation increasing petal numbers (double flower trait) as a 994-bp deletion of the last exon of *Prupe.6G242400* coding for the euAP2/PETALOSA (PET) TOE-type AP2 factor and the deleted region encompasses a miR172 target site (Gattolin et al. 2018, 2020). In addition, the same PET variant was recently proposed to be responsible for slower flower development by controlling temperature-responsive genes in a late flowering peach germplasm (Liu et al. 2024; Cirilli et al. 2021). Although euAP2/PET is primarily involved in flower development, it plays important roles in regulation of gene expression (repressor or activator), hormonal pathways, and environmental responses (Ma et al. 2024). Thus, euAP2/PET could influence peduncle elongation through various direct and indirect mechanisms. These include binding to the promoters of genes involved in cell division and elongation, regulating their expression and consequently influencing peduncle growth. Additionally, it promotes alterations in the levels or sensitivity of cells in the peduncle to growth-promoting hormones such as auxins and gibberellins, resulting in changes in elongation rates. Lastly, it could also help the plant adjust peduncle growth in response to external conditions, ensuring that elongation occurs under favorable conditions.

## Conclusions

In this work, we emphasized the importance of PL and suggested it as a novel trait to be included in breeding programs to reduce mechanical damage at harvest and thereby lower fruit waste. A stable QTL on LG6 was mapped over three years of evaluation, explaining up to 63% of the observed phenotypic variation. Candidate genes within the genetic interval of this stable QTL were identified, and their functional annotations suggest involvement in developmental processes, stress responses, hormone regulation, gene expression regulation, and cellular signaling. The findings reported here provide valuable information for future studies targeting PL and open opportunities for more in-depth investigations using different sources of PL within peach germplasm, validate the QTLs mapped in WxBy progeny, improve mapping resolution and identify genes involved in the regulation of PL in peach.

## Supplementary Information

Below is the link to the electronic supplementary material.

Supplementary file1 (PDF 221 KB)

Supplementary file2 (XLSX 112 KB)

## Data Availability

The datasets presented in this study can be found in online repositories. The names of the repository/repositories and accession number(s) can be found below: www.rosaceae.org.
