# Supplementary material for: A novel trait to reduce the mechanical damage of peach fruits at harvest: The first genetic dissection study for peduncle length
Source: Mol Breed. 2025 Feb 24;45(3):29. doi: 10.1007/s11032-025-01547-3 (PMC11850672; doi:10.1007/s11032-025-01547-3)
Supplement: Supplementary file 1 — Supplementary file1 (PDF 221 KB) [file 11032_2025_1547_MOESM1_ESM.pdf]

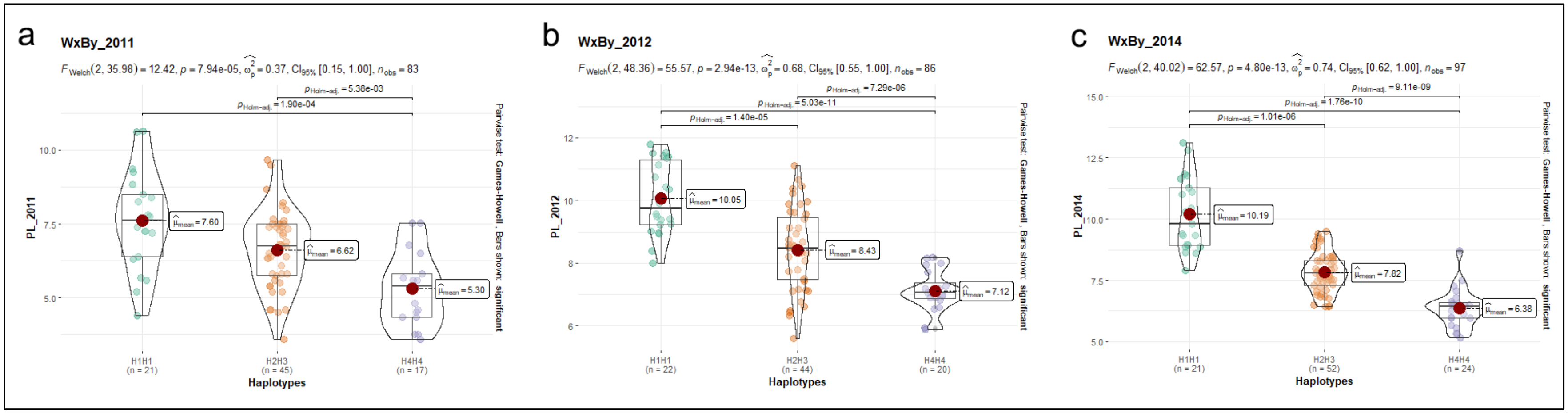

Supplementary Fig. S1: Peduncle length (PL; in mm) in 2011(a), 2012 (b) and 2014 (c) and haplotype effects in the WxBy progeny. Effects were based on the most frequent haplotypes observed for *qPL_WBy_6.1* genetic interval. N represents the number of individuals containing the phenotypic data for PL observed in each haplotype. Significantly different (Games Howell, $p < 0.05$) phenotypic means are identified by bars.
